# Combating Cosmetic Dermatology Misinformation Using Artificial Intelligence

**DOI:** 10.1111/jocd.71196

**Published:** 2026-09-15

**Authors:** Doris Day, Marina Landau, Mohamad Goldust

**Affiliations:** ^1^ Day Dermatology and Aesthetics New York City New York USA; ^2^ Arena Dermatology and Department of Plastic Surgery Shamir Medical Center Be'er Ya'akov Israel; ^3^ Department of Dermatology University of Illinois at Chicago Chicago Illinois USA

**Keywords:** artificial intelligence, cosmetic dermatology, misinformation

Dear Editor,

The rapid expansion of social media has transformed cosmetic dermatology into one of the most visible medical specialties online. Platforms like Instagram, TikTok, YouTube, and Snapchat increasingly shape public perceptions about fillers, neurotoxins, laser procedures, skin rejuvenation, and anti‐aging therapies. While this digital visibility enhances patient engagement and awareness, it has also accelerated the spread of misinformation, unrealistic expectations, unsafe “do‐it‐yourself” procedures, and promotion of unverified aesthetic treatments [1]. Artificial intelligence (AI) may offer a crucial solution to combat misinformation in cosmetic dermatology and improve digital health communication.

Social media platforms have widely circulated content promoting self‐injection of hyaluronic acid fillers, high‐concentration at‐home chemical peels, and microneedling, potentially resulting in vascular occlusion, burns, scarring, hyperpigmentation, and infections. Beauty filters and AI‐enhanced facial editing may further create unrealistic aesthetic expectations, encouraging patients to pursue procedures based on digitally altered appearances rather than achievable clinical outcomes.

Misinformation frequently originates from non‐medical influencers, masked marketing, and edited or AI‐generated visual content exaggerating treatment outcomes. Deepfakes, image filters, and manipulated before‐and‐after photographs can distort patient expectations, particularly among adolescents and young adults. The speed and scale of misinformation spreading through social media generate traditional fact‐checking approaches insufficient [2]. Unlike more clearly falsifiable content encountered in fields such as image forensics, cosmetic dermatology presents a particular challenge: the boundary between acceptable promotional content, subjective aesthetic claims, exaggeration, and potentially harmful misinformation may be unclear, making specialty‐specific training and expert validation of AI models essential.

AI‐powered systems could help identify misinformation through automated monitoring of dermatologic content. Natural language processing algorithms can analyze posts, captions, and comments for misleading claims, while computer vision technologies can identify manipulated aesthetic images and unrealistic treatment outcomes. AI‐based facial analysis, aesthetic simulation, and image‐processing technologies are already used within aesthetic medicine, demonstrating the feasibility of applying AI to cosmetic images. However, these technologies may also reinforce unrealistic beauty standards, highlighting AI's potential both to generate and to counter misleading aesthetic content.

Large language models could additionally support evidence‐based patient education by providing information about expected outcomes, risks, contraindications, and post‐procedural care. Real‐time AI surveillance could identify rapidly spreading cosmetic trends and enable professional societies to issue timely corrective guidance [3].

These challenges exist within an evolving social‐media governance environment. Australia's introduction of a minimum social‐media age of 16 demonstrates increasing societal regulation of online exposure, while recent shifts from traditional third‐party fact‐checking toward community‐based moderation illustrate a contrasting approach. These developments raise an important question: should responsibility for medical misinformation rest primarily with social‐media companies, health professionals, regulators, users, or a shared governance model?

AI systems nevertheless may propagate inaccuracies when trained on biased or low‐quality datasets, while generative AI may produce fabricated or oversimplified medical information. Automated moderation also raises concerns regarding censorship, transparency, privacy, and algorithmic bias. Dermatologist oversight could be implemented through reference datasets curated by professional dermatology societies, expert review of high‐risk flagged content, periodic auditing of algorithms, and rapid‐response groups reviewing viral cosmetic claims. AI should therefore serve as a scalable screening tool rather than the final arbiter of medical truth [4].

Dermatologists themselves are also participants in this evolving digital ecosystem. Teledermatology, virtual aesthetic consultations, remotely supervised treatment, and physician‐recommended home‐care regimens increasingly blur the distinction between clinic‐based and consumer‐directed cosmetic care. The relevant distinction may therefore be not simply “at home” versus “in clinic,” but whether treatment is evidence‐based, appropriately selected, professionally supervised, and accompanied by adequate follow‐up. Further studies should quantify how frequently cosmetic dermatologists provide such remote care and whether its use is increasing [5].

Ultimately, consumer‐facing cosmetic AI applications should be distinguished from clinically validated medical tools. Improving AI literacy among clinicians and patients is therefore critical. Because cosmetic misinformation often falls within a gray area rather than being clearly true or false, effective AI implementation will require dermatologist‐led validation, transparent governance, collaboration between professional societies and digital platforms, and ongoing digital education.

## Author Contributions

The authors have nothing to report.

## Funding

The authors have nothing to report.

## Ethics Statement

This article does not involve human participants or animals.

## Conflicts of Interest

The authors declare no conflicts of interest.

## Data Availability

Data sharing not applicable to this article as no datasets were generated or analysed during the current study.
